# Promoting reproducibility with Code Ocean

**DOI:** 10.1186/s13059-021-02299-x

**Published:** 2021-02-19

**Authors:** Barbara Cheifet

**Affiliations:** Springer Nature, New York, NY USA

*Genome Biology* has built a reputation in the community for our strong data deposition policies [1]. We require our published methods and software to have their source code deposited on a public software repository, such as Github, and in a DOI-assigning repository, such as zenodo. The source code must also be released under an open source license compliant with OSI, and this must be clearly stated on the repository and in the manuscript. Not only is it essential to have code available for reproducing results, but it enables other researchers to build on the work or find potential pitfalls of a method.

As editors, we assume that our reviewers are looking at source code to some extent; however, it is difficult to determine how thoroughly a code has been analyzed. We recognize that reviewing code can be cumbersome, as authors must first make sure that code is accessible for reviewers to check, and reviewers must then be able to download the code and data and make sure that they have the correct dependencies installed to be able to confirm the reproducibility of the results. In addition, a reviewer's identity could be revealed unintentionally if downloading a software from a page controlled by the author(s).

Recently, several *Nature* journals, including *Nature Methods*, *Nature Biotechnology*, and *Nature Machine Intelligence* completed a trial with Code Ocean, a cloud-based reproducibility platform that aims to help reviewers and authors facilitate peer review of source code [2, 3]. This trial has since been made permanent and expanded to other journals at Springer Nature, including *BMC Bioinformatics* and *Scientific Data*. *Genome Biology* is excited to announce that at the end of 2020, we also have partnered with Code Ocean, with the aim of making the peer review of source code easier for both reviewers and authors.

Code Ocean is based on Docker and serves as a platform for the deposition of all code and data necessary for reproducing analysis in the cloud. The platform integrates the source code, metadata, and dependencies into one “capsule” within a single, user-friendly web interface. By doing so, it ensures that all data required for re-analysis is available and that reviewers have easy access to it from any set-up or personal computer. It also provides a check that the code supplied compiles and runs. Users and reviewers also have the ability to upload their own data for testing or to experiment themselves with parameter options. Code Ocean provides a DOI for citation and also has partnered with CLOCKSS to ensure that capsules are archived and preserved. Capsules are also tested to make sure that they function correctly.

At *Genome Biology*, many of our authors submit their methods to the journal having already submitted their source code to Github and zenodo. These authors may feel that the creation of a Code Ocean capsule is redundant, and we would agree that this is a fair point. However, we would like the authors to consider creating a Code Ocean capsule as an alternative to submitting source code to both Github and zenodo.

As expectations for peer reviewers become higher, and more and more studies are being shown to be difficult to reproduce, better standards are needed to ensure that code is being reviewed effectively and completely. With the creation of a capsule in Code Ocean, editors are also able to monitor if reviewers have accessed the code for review, ensuring that at least one reviewer has done so. If the capsule has not been accessed, the editor can then check back in with the reviewer(s) to ask that they do so. The trials performed at the other *Nature* journals showed that reviewers were verifying the code and reproducing the results.

For now, *Genome Biology* will be suggesting deposition of source code to a Code Ocean capsule at peer review stage for any paper with novel source code. Of course, we expect that authors who have already deposited to Github or zenodo may decline to also deposit to Code Ocean, but we hope that as more authors become aware of this platform, more papers will start coming to us with Code Ocean capsules already created.

We appreciate that Code Ocean is pushing authors to make their code available for review, upholding FAIR standards, and promoting reproducibility in bioinformatics, while also making it easy for reviewers to access and analyze code and data. We hope that our authors and reviewers will be happy to make use of this service and continue to work towards greater reproducibility and accountability in science.
